# Development the Hydrophobic Property of Polyvinyl Alcohol/Silicon Dioxide/Titanium Dioxide Nanocomposites for Self-Cleaning and Soil Stabilization

**DOI:** 10.3390/molecules30081664

**Published:** 2025-04-08

**Authors:** Rania F. Khedr, Mohamed Abd Elhady

**Affiliations:** 1 Chemistry Department, Al Leith-University College, Umm Al-Qura University, Makkah 24382, Saudi Arabia; 2 National Center for Radiation Research and Technology (NCRRT), Department of Polymer Chemistry, Egyptian Atomic Energy Authority (EAEA), Cairo 11787, Egypt

**Keywords:** polyvinyl alcohol, silicon dioxide, titanium dioxide, nanocomposite, self-cleaning, soil stabilization

## Abstract

This study focused on synthesizing polyvinyl alcohol (PVA) utilizing glutaraldehyde (GA) as a crosslinking agent and silicon dioxide (SiO_2_) nanopowder with titanium dioxide (TiO_2_) nanopowder to reduce or prevent the hydrophilic property of PVA. Integrating SiO_2_ and TiO_2_ into the PVA boosted the hydrophobicity, thermal properties, and self-cleaning of the PVA film. The characteristic properties of PVA/GA, PVA/SiO_2_/GA, and PVA/SiO_2_/TiO_2_/GA nanocomposites polymer membranes were investigated by gel content, swelling capacity, Fourier transform infrared (FTIR) spectroscopy, X-ray diffraction patterns (XRD), scanning electron microscope (SEM), thermal gravimetric analysis (TGA), and contact angle. The resulting PVA/5%SiO2/1%TiO_2_/GA nanocomposite exhibits much better physical properties than PVA/GA hydrogel (water absorbency from 3.1 g/g to 0.07 g/g and contact angel from 0° to 125°). In addition, the nanocomposite retains very low swelling properties. These prepared nanocomposites are promising in a variety of applications such as sand soil stabilizers, construction, and building works where they exhibit excellent water resistance performance. This study introduces a novel approach for creating hydrophobic polymeric membranes from hydrophilic polymeric materials to stabilize sandy soil effectively.

## 1. Introduction

Polyvinyl alcohol (PVA), a biodegradable polymer, is widely recognized for its water solubility and ability to form films. In recent years, three-dimensional network PVA hydrogels constructed from crosslinked chain networks (physical or chemical) have gained significant interest due to their superior biocompatibility and applicability, making them suitable for diverse fields such as agriculture, biomedical engineering, food packaging, separation and purification, catalytic and electrochemical applications, and adsorption chemistry [1,2,3]. The diverse applications of PVA stem from its unique hydrogen bonding, both within and between its molecular chains, which influences the gel’s structure and mechanical properties [4,5]. These bonds can be modified through chemical or physical crosslinking. Environmental factors further impact the gel’s swelling behavior, osmotic characteristics, and mechanical strength, all of which depend on the crosslinking method and extent [6,7]. Among the polymers, PVA stands out as a highly appealing polymer because it is thermally and environmentally stable, non-toxic, water-soluble, and exhibits excellent film-forming ability. PVA features a backbone carbon chain with attached hydroxyl groups, as its O-H bonds help to form polymer nanocomposite. PVA’s remarkable resistance to chemicals and strong physical characteristics make it highly suitable for various uses in construction, including geotextiles [8], wallpaper adhesives [9,10], and cement chemical stabilizers [11,12]. Additionally, various PVA geocomposites have been explored, including those incorporating ground rice husk [13], high-calcium fly ash [14], wheat straw [15], and butane tetracarboxylic acid (BTCA) [16]. These geocomposites are utilized in earthen construction as surface pretreatment agents for blocks and as fiber reinforcements within cement-based composites. The hydroxyl groups in PVA can modify surface adhesion characteristics among blocks, matrices, and reinforcing fibers [17,18]. Incorporating PVA into cement matrices helps minimize the development of inoculated structures caused by cement particles and reduces the bleeding effect in cement paste [19,20]. The material’s physical characteristics also regulate polymerization and hydrolysis levels, enhancing water resistance, seepage management, and chemical stability in soil. Furthermore, PVA is non-toxic, biodegradable, and biocompatible, positioning it as an eco-friendly and sustainable option for construction applications [21,22]. To improve the durability and functionality of PVA films, various chemical modification techniques have been applied, including crosslinking [23], electrospinning [24,25], blending [26,27], sol–gel methods [28,29], and grafting [30]. Crosslinking serves as an effective method for modifying both the chemical and physical characteristics of films. The presence of multiple hydroxyl groups in PVA enables straightforward crosslinking with diverse multifunctional compounds that react with -OH groups, facilitating the formation of three-dimensional networks. Improving PVA properties can be done by introducing other functional materials. Inorganic binary nanomaterials composed of TiO_2_ and SiO_2_ have attracted much attention from researchers [31,32,33] and are extensively employed as antireflection coatings, optical-chemical sensors, glass, and catalyst supports due to their exceptional thermal and optical characteristics. TiO_2_ and SiO_2_ nanoparticles are more efficient in improving the thermal and mechanical properties of polymeric materials than using either one alone [34]. Binary inorganic nanomaterials are defined as mixtures of two components at the molecular or nanoscale level. Many natural materials contain inorganic building blocks distributed at the nanoscale. Optimal combination can improve thermal and chemical stability, thermosensitivity, mechanical strength, and optical, thermal, magnetic, electrical, anti-corrosion, and fire resistance properties, as well as alter the properties of polymers such as PVA. This makes the properties of binary inorganic nanomaterials superior to those of their counterparts [35]. PVA/SiO_2_/TiO_2_ binary inorganic nanomaterials are a type of nanocomposite that has received significant attention from scientists in the field of materials science. These materials exhibit unique properties that make them suitable for a wide range of applications, including drug delivery systems, coatings, and sensors. The addition of SiO_2_ and TiO_2_ nanoparticles to a PVA matrix enhances the mechanical properties of the nanocomposite, including improved hydrophobicity. The enhancement of the polymer matrix is due to the strong surface interaction between the nanoparticles and the PVA chains, which results in stress transfer from the polymer matrix to the nanoparticles [36]. Therefore, it is very important to fabricate PVA films with improved physical properties [37]. Therefore, extensive efforts have been devoted to incorporating inorganic materials into PVA. The mechanical performance of PVA can be significantly enhanced through its interaction with inorganic materials [38]. Silicon dioxide (SiO_2_) nanopowder is a commonly used reinforcing filler in thermoplastic polymers, known for enhancing mechanical performance [39]. The presence of silanol groups (Si-OH) in SiO_2_ enables strong interactions with the polar functional groups of polymers [40]. Due to this property, it is frequently employed as a strengthening additive to improve the mechanical characteristics of thermoplastic materials [41]. Recently, silica particles with good chemical stability, toughness, non-cytotoxicity, and multifunctionality have stimulated research interest in the fabrication of polymer-material nanocomposites [42]. Researchers have prepared moldable polymer-material nanocomposites through interactions between polymer and silica. Titanium dioxide (TiO_2_) nanopowders are a key metal oxide, widely utilized as a catalyst and a material used for removing and absorbing metals or dyes. TiO_2_ is extensively utilized for its cost-effectiveness and exceptional ability to break down pollutants. Its unique optical characteristics make it suitable for diverse industrial uses, including environmental cleanup to eliminate contaminants, solar energy conversion, photovoltaic systems, and photocatalytic coatings [43]. Enhancing the efficiency of titanium dioxide is crucial for its broader application, and this can be achieved by combining titanium dioxide with another metal oxide, such as silicon dioxide. Both titanium dioxide and silicon dioxide materials provide a larger surface area, which allows for improved application performance. TiO_2_ is a preferred choice for enhancing membranes [43] because of its exceptional photocatalytic properties, extensive surface area, adaptable surface functionalities, and durability under UV radiation [44]. The addition of SiO_2_ and TiO_2_ to the PVA polymer matrix enables the creation of PVA/SiO_2_/TiO_2_ nanocomposite films through the sol–gel method [45]. Significantly, the addition of solid SiO_2_ and TiO_2_ fillers to the PVA solution matrix helps minimize or entirely inhibit the swelling ratio of the resulting PVA/SiO_2_/TiO_2_ nanocomposite film. The thermal and mechanical characteristics, along with dimensional stability, can be enhanced. In this study, a nanocomposite polymer film was created by combining SiO_2_ and TiO_2_ solutions with PVA through a sol–gel process involving hydrolysis and condensation steps. A 1% glutaraldehyde (GA) was introduced directly into the PVA polymer solution to facilitate the crosslinking process. SiO_2_ and TiO_2_ particles serve as solid softeners, which can enhance the chemical and thermal properties, hydrogen bonding interactions between SiO_2_ and PVA, and the homogeneous interconnected network structure, and enhance the PVA/SiO_2_/TiO_2_ nanocomposite polymer film’s stability for use as a stabilizer for sandy soil by mixing or spraying on the surface of sandy soil.

## 2. Results and Discussion

### 2.1. Gel Content Study

**Scheme 1:**
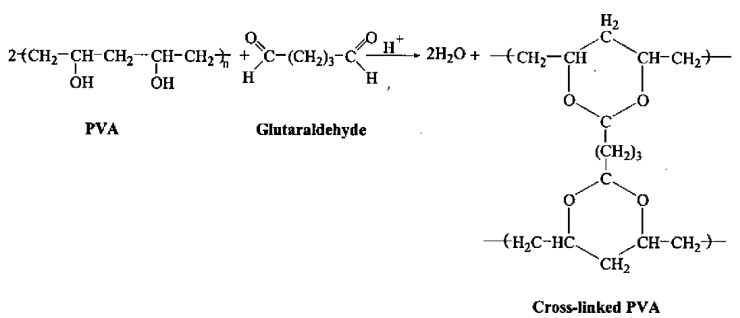
Reaction of PVA with glutaraldehyde.

**Figure 1:**
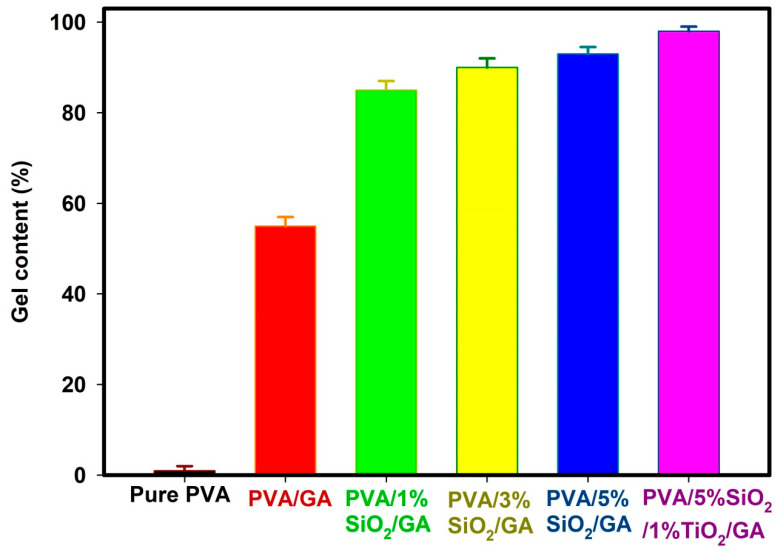
Effect of glutaraldehyde, SiO_2_, and TiO_2_ on gel content of PVA.

Gel content reflects the extent of crosslinking within a hydrogel. Increased crosslinking results in a more rigid structure, diminishing the hydrogel’s water absorption capacity [46]. Figure 1 demonstrates how gel content (%) varies with the addition of glutaraldehyde, SiO_2_ and TiO_2_ to the PVA. The gel content (%) increases progressively with the incorporation of glutaraldehyde, SiO_2_ and TiO_2_ (PVA/GA, PVA/1, 3, 5%SiO_2_/GA and PVA/5%SiO_2_/1%TiO_2_/GA) peaking at a stable maximum of (98.7%) for PVA/5%SiO_2_/1%TiO_2_/GA. The optimum miscibility and high gel content (%) formation was found to be at PVA/5%SiO_2_/1%TiO_2_/GA. Increased gel content and crosslinking density result in reduced water absorbency. Various elements, including polymer composition, nanocomposites, and radiation dosage, influence the gel content of polymers. On the other hand, with the increase of SiO_2_ content (1, 3, 5%) on PVA/GA, as well as the presence of 1% TiO_2_ (PVA/5% SiO_2_/1% TiO_2_/GA), the gel content increases, peaking at 98.7%. The reaction of PVA with aldehydes is poly(vinyl acetal) resins [13]. The formation of PVA gel by dialdehyde (glutaraldehyde) is shown as follows [47] (Scheme 1).

### 2.2. Swelling Behavior

**Figure 2:**
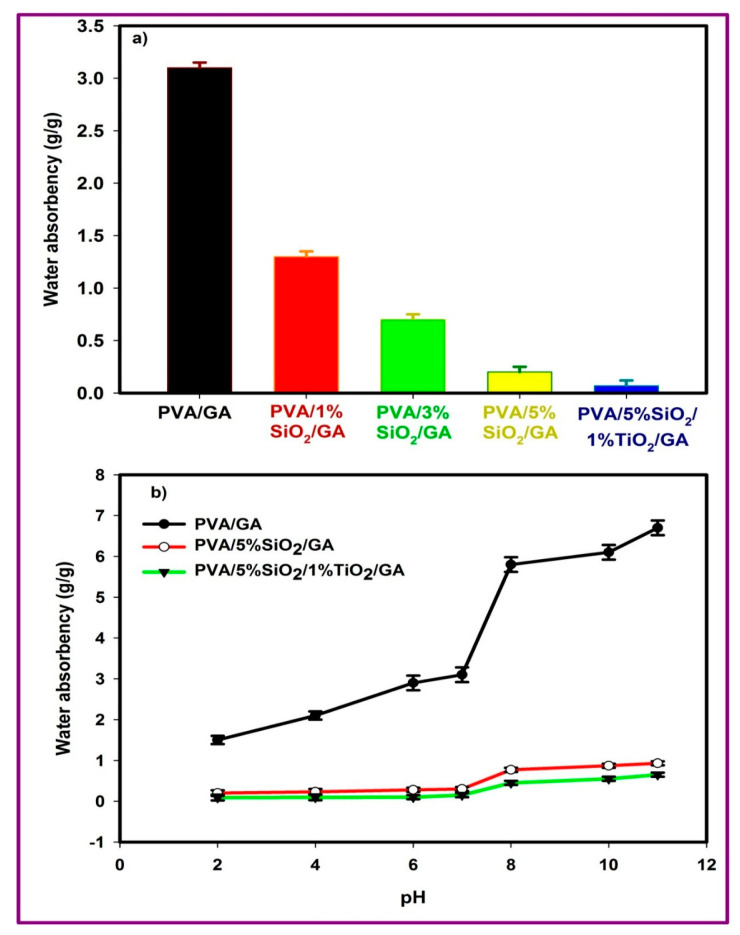
(**a**) Effect of SiO_2_ and TiO_2_ on water absorbency (g/g) of PVA/GA (**b**) Effect of pH on water absorbency (g/g) of PVA/GA, PVA/5%SiO_2_/GA, and PVA/5%SiO_2_/1%TiO_2_/GA.

The swelling value indicates the proportion of water or solution absorbed by the hydrogel relative to its dry mass. In the swelling experiment, the hydrogel was immersed in distilled water for a time interval of 24 h. The swelling ratio reflects the level of crosslinking within the polymer. Higher crosslinking density reduces the swelling ratio, as fewer gaps in the network are available for solvent absorption. Figure 2 illustrates the impact of varying concentrations of SiO_2_ and TiO_2_ nanopowders on PVA/GA water absorption. The PVA/GA water absorption declined from 3.1 g/g to 0.07 g/g due to the presence of SiO_2_ and TiO_2_ at 5%SiO_2_ and 1%TiO_2_ (PVA/5% SiO_2_/1% TiO_2_/GA), and this ratio is the best ratio as PVA/GA turns from hydrophilic to hydrophobic, as shown in Figure 2. This outcome occurred because the crosslinking density rose as the SiO_2_ concentration increased from 1% to 5%, combined with the inclusion of 1% TiO_2_. The greatest gel content was achieved with PVA/5% SiO_2_/1% TiO_2_/GA, indicating optimal crosslinking within the polymer chain at these concentrations. Additionally, the crosslinking density rose as the SiO_2_ content in the PVA/GA mixture increased. This is attributed to the formation of covalent bonds between the polymer chains (PVA/GA) and the added SiO_2_ and TiO_2_ nanopowders. As a result, the hydrophobic properties of polyvinyl alcohol were significantly enhanced. The formation of covalent bonds between PVA/GA chains and SiO_2_ and TiO_2_ nanopowders occurred as the particles restricted the polymer chains’ mobility owing to the bonding between the particles and the polymer chains (Si-O-PVA-O-Ti) [48]. Higher crosslinking in the polymer chains caused the segments to contract, reducing the hydrogel’s water absorption capacity. This occurred because the tighter network left less room for free water to occupy the available spaces.

To study the effect of acidic and basic media on the swelling behavior of the samples (PVA/GA, PVA/5% SiO_2_/GA, and PVA/5% SiO_2_/1% TiO_2_/GA), the samples were immersed at different pH values for 24 h, as shown in Figure 2b. Figure 2b shows that the swelling ratio of the samples at pH lower than 7 decreased significantly, especially for the nanocomposites containing SiO_2_ and TiO_2_ (PVA/5% SiO_2_/GA and PVA/5% SiO_2_/1% TiO_2_/GA). It was observed that the nanocomposites increased the crosslinking density of the PVA/GA hydrogel, and as a result, the pore size between the network chains decreased, resulting in a lower swelling ratio for the hydrogels containing SiO_2_ and TiO_2_ [49,50,51]. On the other hand, it is clear that the swelling ratio of the hydrogels was strongly affected by the pH of the swelling medium, and increased significantly with increasing solution pH. For example, it can be seen from Figure 2b that the swelling ratio of PVA/GA increased from 1.5 to 3.1 (g/g) as the pH of the buffer solution was raised from 2 to 7, reaching a maximum value of 6.7 at pH 11. Similar observations were also made for the PVA/5% SiO_2_/GA and PVA/5% SiO_2_/1% TiO_2_/GA nanocomposites. With increasing pH values (pH = 2–7), the swelling ratio increased from 0.1 to 0.23 (g/g) for the PVA/5% SiO_2_/GA nanocomposite and from 0.03 to 0.07 (g/g) for the PVA/5% SiO_2_/1% TiO_2_/GA nanocomposite, respectively. At pH 11, this suggests that SiO_2_ and TiO_2_ nanoparticles can be considered additional crosslinking points in hydrogel (PVA/GA), resulting in a decrease in the swelling capacity of the nanocomposites [52]. This can be explained by the fact that at pH below 7, hydrogen bonding in PVA/GA decreases because the acidic environment may ionize the hydroxyl groups, reducing the ability of PVA to interact with water, resulting in a decrease in swelling. Under alkaline conditions (pH > 7), swelling may increase due to the ionization of hydroxyl groups, which enhances water absorption. The presence of silicon dioxide (SiO_2_) and titanium dioxide (TiO_2_) nanoparticles in the PVA/GA matrix played an important role in the pH-sensitive swelling behavior. The stability of the nanocomposites was observed at pH levels below 7, and their swelling increased with increasing pH to a basic state, as shown in Figure 2b. The limited swelling results from the decomposition of some of the SiO_2_ and TiO_2_ nanoparticles covering the polymer matrix (PVA/GA), which leads to the deprotonation of the hydroxyl groups (-OH), resulting in stronger hydrophilic interactions. The swelling ratio of the nanocomposite hydrogel was significantly lower compared to PVA/GA, due to the presence of silicon dioxide and titanium dioxide nanoparticles, which prevent swelling of the hydrogel matrix [53,54]. Conclusion: Optimal stability was observed at a neutral pH (~7). Limited swelling occurs in alkaline environments for the nanocomposites, making them effective in withstanding external environmental conditions and highly effective in many environmental applications, especially in stabilizing sandy soils.

### 2.3. FTIR Spectra

**Figure 3:**
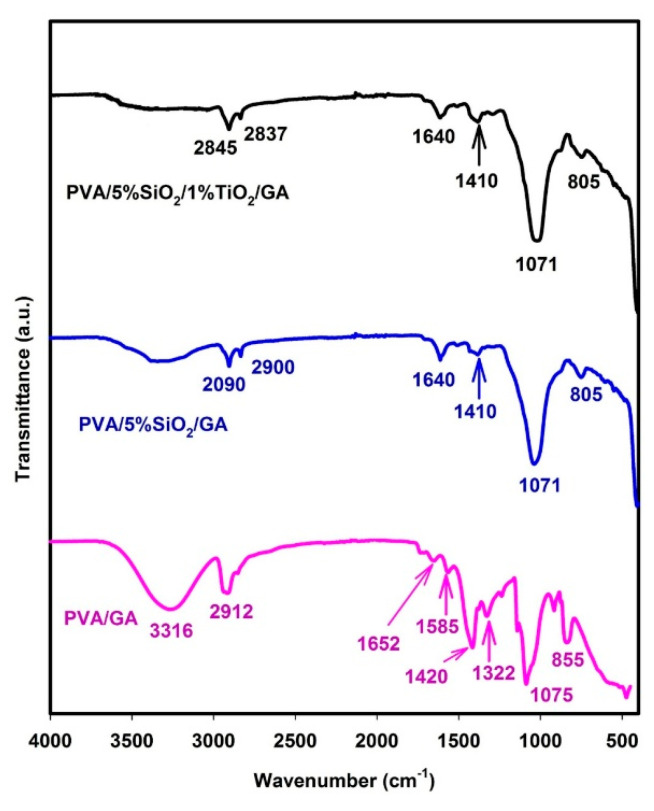
FTIR spectra of PVA/GA, PVA/5%SiO_2_/GA, and PVA/5%SiO_2_/1%TiO_2_/GA.

The compound was characterized using infrared spectroscopy, and the interactions within the polymer matrices were thoroughly examined. Using the technique of Fourier transform infrared (FTIR) spectroscopy, a comparison was performed among the spectra of PVA/GA, PVA/5%SiO_2_/GA, and PVA/5%SiO_2_/1%TiO_2_/GA nanocomposites, which is shown in Figure 3. A peak at 3316 cm^−1^ corresponds to the O–H stretching vibration in PVA/GA, reflecting specific interactions within the polymer network. The absorption at 2912 cm^−1^ is attributed to the C–H stretching of CH_2_, with the peak specifically indicating sp^3^ C–H stretching in aldehydes, accompanied by a shoulder peak from the alkyl chain. This confirms acetal bridge formation due to PVA and GA crosslinking. Peaks at 1652 cm^−1^ and 1585 cm^−1^ are linked to C=O stretching vibrations. The CH_2_ group’s wagging vibration appears at 1322 cm^−1^, corresponding to C–H deformation at lower wavenumbers. Additionally, C–C stretching is observed at 1245 cm^−1^, while C–O stretching occurs at 1075 cm^−1^. A peak near 855 cm^−1^ is associated with the C–H rocking mode in PVA/GA. Figure 3 compares the FTIR spectra of PVA/GA with PVA/5%SiO_2_/GA and PVA/5% SiO_2_/1%TiO_2_/GA nanocomposite. The band at 3316 cm^−1^ decreased υ diminished in intensity with 5% SiO_2_ content and disappeared with 5% SiO_2_/1% TiO_2_. Meanwhile, a slight increase and shift in the intensity of the band at 1652 cm^−1^ to 1640 cm^−1^ were noted, specifying a reduction in the material’s hydrophilic nature. These findings clearly demonstrate the successful formation of an organic–inorganic nanocomposite network composed of PVA, 5%SiO_2_, and 1%TiO_2_. On the other hand, the shift from 1652 cm^−1^ to 1640 cm^−1^ is evidence of the formation of hydrogen bonds between SiO_2_ and TiO_2_ with PVA. When this type of interaction occurs, the peak may shift to lower frequencies (1640 cm^−1^) as a result of increased interactions between molecules, which lead to a decrease in the ability of PVA to absorb water. Two significant peaks at ν = 2909–2900 cm^−1^ and ν = 2845–2837 cm^−1^ could be observed for C-H stretching linked to aldehyde groups [55]. GA functioned as a crosslinker, connecting PVA polymer chains with an organic–inorganic hybrid binder. On the other hand, no peaks were observed within the range 3630–3200 cm^−1^, indicating that all -OH groups were bound [55]. Additionally, the Si-O- and Ti-O- stretching frequency peaks at 805 and 790 cm^−1^ showed a slight shift towards a higher wavenumber in comparison to PVA/GA [56]. This shift is likely due to covalent interactions between PVA/GA polymer and SiO_2_ and TiO_2_. The IR spectrum of the PVA/GA composite shows a band at 1420 cm^−1^, which diminishes in intensity and shifts to 1410 cm^−1^ in the nanocomposite film [57]. This alteration suggests that the vibrations associated with this band become decoupled, likely due to interactions between the TiO_2_ nanoparticles and the O-H groups derived from the PVA/GA polymer chains. Moreover, the peak at 1071 cm^−1^ assigned to Si-O- stretching vibrations showed the establishment of covalent bonds between the organic and inorganic phases. Compared to the PVA/GA IR spectra, the peak of -OH group stretching vibrations shifted to a lower wavenumber. This shift suggests that a significant portion of the hydroxyl groups in PVA interacted with the decomposition products of SiO_2_ and TiO_2_, leading to the formation of hydrogen bonds.

### 2.4. X-Ray Diffraction Patterns

**Figure 4:**
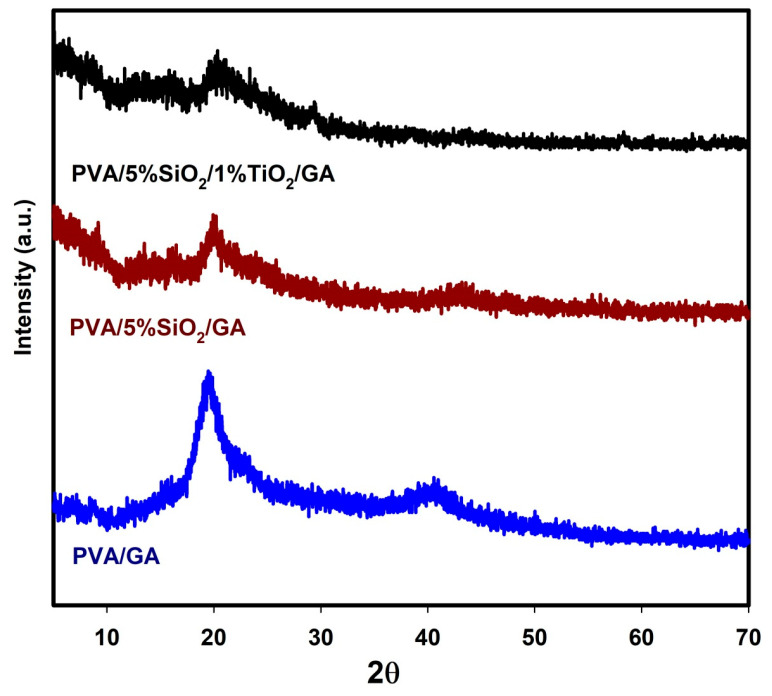
X-ray diffraction patterns of PVA/GA, PVA/5%SiO_2_/GA, and PVA/5%SiO_2_/1%TiO_2_/GA.

X-ray diffraction patterns are highly effective for analyzing structural and morphological changes, as well as assessing the crystallinity of nanocomposite films of PVA/GA with the SiO_2_ and TiO_2_ nanopowders. According to Figure 4, a sharp diffraction peak around 2θ = 19.45° appeared, which aligns with the (101) plane of the semi-crystalline structure of PVA/GA in the PVA chain [31]. Nevertheless, in the samples incorporating 5%SiO_2_ and 5%SiO_2_/1%TiO_2_, this peak broadens significantly and shifts, resulting in flatter and less intense diffraction patterns. The PVA/GA membrane exhibited a high degree of crystallinity, primarily attributed to the presence of hydroxyl groups in its side chains. In contrast, for the PVA/5% SiO_2_/GA and PVA/5% SiO_2_/1% TiO_2_/GA nanocomposites, the hydroxyl groups of the PVA molecules interacted with SiO_2_ or SiO_2_/TiO_2_ particles. This interaction led to a reduction or complete elimination of hydroxyl groups, ultimately causing a decline in crystallinity. On the other hand, the insertion of SiO_2_ and TiO_2_ into the PVA/GA matrix induced significant shifts and a reduction in both peak positions and intensities, demonstrating the successful integration of these fillers into the nanocomposite polymer matrices. The main peak (2θ = 19.45°) shifted to 19.8–20.1° and reduction in the intensity, indicating the influence of SiO_2_ and TiO_2_. This occurs because the nanoparticles interfere with PVA chain packing, reducing crystallite formation.

### 2.5. HRTEM and SEM Analysis

**Figure 5:**
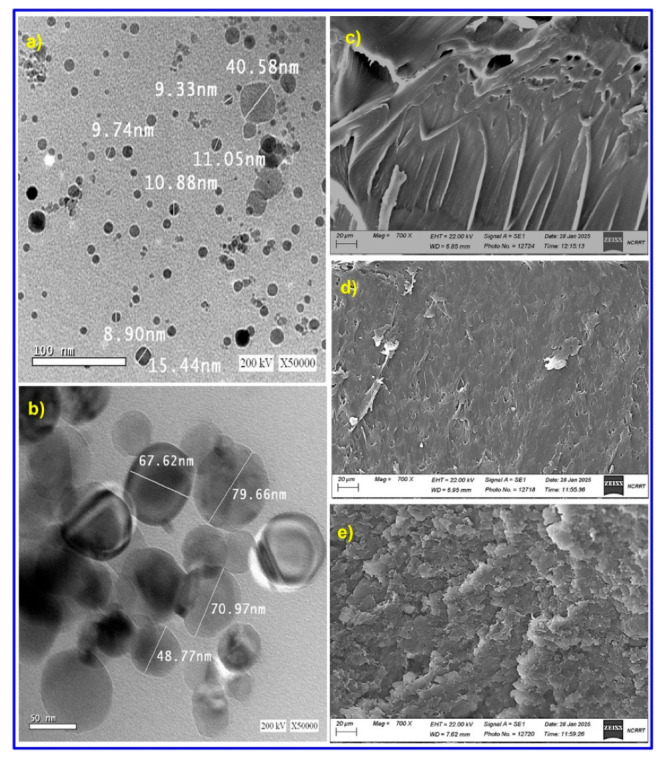
(**a**) HRTEM image of SiO_2_; (**b**) HRTEM image of TiO_2_; (**c**) SEM of PVA/GA; (**d**) SEM of PVA/5%SiO_2_/GA nanocomposites; (**e**) SEM of PVA/5%SiO_2_/1%TiO_2_/GA nanocomposites.

High-resolution transmission electron microscopy (HRTEM) is one of the most valuable techniques for directly analyzing the structure, size, and shape of synthesized nanoparticles. Figure 5a,b show high-resolution transmission electron microscopy (HRTEM) images of silicon dioxide (SiO_2_) and titanium dioxide (TiO_2_). The size of the silicon dioxide nanoparticles ranged from 8.9 to 40.58 nm, with an average size of approximately 15.44 nm. The titanium dioxide nanoparticles appeared nearly spherical, with sizes ranging from 48.77 to 79.66 nm. The average particle size was 67.5 nm.

Scanning electron microscope (SEM) is an advanced imaging tool that generates detailed, high-resolution pictures by directing a concentrated electron beam across a specimen. It is widely used to analyze the surface structure and composition of materials at a very fine scale. The morphological difference between PVA/GA and the PVA/GA nanocomposites with 5%SiO_2_ and 5%SiO_2_/1%TiO_2_ contents was analyzed by cross-sectional SEM images (Figure 5c–e). The surface morphology of PVA/GA, PVA/5%SiO_2_/GA, and PVA/5%SiO_2_/1%TiO_2_/GA films was inspected utilizing SEM analysis. As shown in Figure 5c, the morphology of PVA/GA hydrogel is different from that of 5%SiO_2_ and 5%SiO_2_/1%TiO_2_ nanocomposites. The cross-section of PVA/GA contains some obvious large pores, looks more compact and rougher, and shows expanded and porous networks. The distribution of SiO_2_ and TiO_2_ particles was homogeneous and uniform at the optimum concentration (i.e., 5 wt% SiO_2_ and 1 wt% TiO_2_). When TiO_2_ was loaded at 1 wt%, the particles were observed to be inserted within the membrane pores, which reduced or covered the membrane porosity, as shown in Figure 5e. It can be observed that the loading of TiO_2,_ in addition to SiO_2,_ enhanced the PVA/GA surface roughness. The SEM analysis reveals that the surface of the film exhibits sand-like and pebble-like structures, confirming the increased surface roughness with the presence of TiO_2_ and the adhesion of TiO_2_ particles to the PVA/GA film’s surface. The SEM images depicted in Figure 5c–e reveal that the PVA/5%SiO_2_/1%TiO_2_/GA composite exhibits a uniform distribution of 5%SiO_2_ and 1%TiO_2_ within the PVA matrix, with no visible cracks or voids surrounding the particles. In contrast to the PVA/GA membrane, the surface of the PVA/5%SiO_2_/1%TiO_2_/GA membrane appears significantly rougher and more compact. Moreover, the SiO_2_ and TiO_2_ were a macroscopic homogeneous system in the PVA/GA matrix.

### 2.6. Thermal Analyses

**Figure 6:**
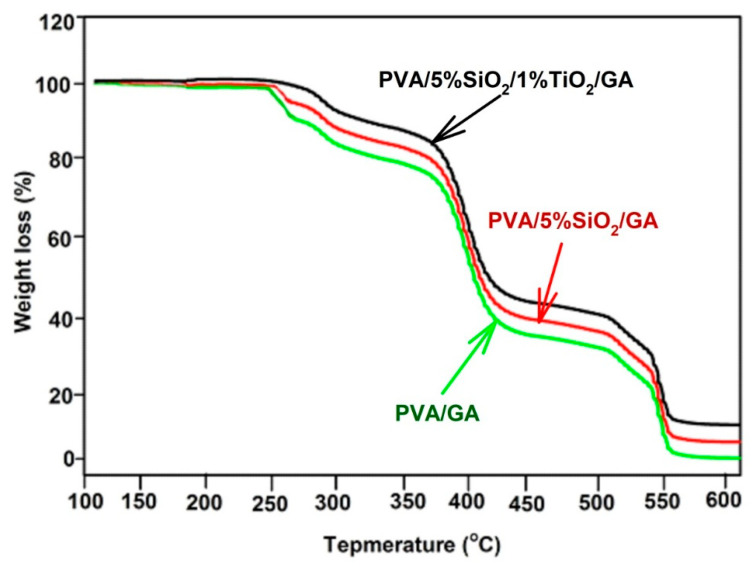
TGA curves for PVA/A, PVA/5%SiO_2_/GA, and PVA/5%SiO_2_/1%TiO_2_/GA nanocomposites.

TGA analysis provides a direct indicator of thermal stability and decomposition by measuring the mass reduction of a material relative to temperature changes. In Figure 6, the TGA profiles for PVA/GA, PVA/5% SiO_2_/GA, and PVA/5% SiO_2_/1% TiO_2_/GA nanocomposites are illustrated. The thermal decomposition process occurs in three distinct stages across temperature ranges of 245 °C to 375 °C, 375 °C to 425 °C, and 425 °C to 540 °C. The most significant mass reduction was observed during the initial phase, between 245 °C and 375 °C. In this stage, the PVA/GA material begins to break down, followed by subsequent degradation in the second and third stages. The structural breakdown of PVA/GA within the 375 °C to 545 °C range is attributed to the fragmentation of the polymer film’s backbone. By the time the temperature reaches 545 °C, nearly 99% of the material’s weight is lost. As observed from this figure, the residual weights of PVA/5% SiO_2_/GA and PVA/5% SiO_2_/1% TiO_2_/GA nanocomposite films are significantly higher compared to PVA/GA film, attributed to inserting SiO_2_ and TiO_2_ in the polymer matrix. The three stages of decomposition correspond to the removal of side groups in the lower temperatures and the disintegration of the PVA backbone in the higher temperatures, respectively [58,59]. The internal SiO_2_ and TiO_2_ in the PVA/GA matrix reduced the decomposition temperature of the PVA/GA film, which was caused by the fragmentation of the C\C backbone in the PVA polymer structure, resulting in a total weight loss of approximately 75–82 wt% at 540 °C. This phenomenon can be attributed to the robust interaction between SiO_2_, TiO_2_, and the PVA/GA polymer chains. Incorporating SiO_2_ and TiO_2_ reduces the extensive hydrogen bonding network within the PVA/GA chains, thereby enhancing the material’s thermal stability [58,60]. Based on the TGA results, the addition of SiO_2_ and TiO_2_ in the PVC matrix alters the thermal stability and decomposition of the PVA/GA film to values 100 °C lower than the temperature. In general, the degradation peak of PVA/5%SiO_2_/GA and PVA/5%SiO_2_/1%TiO_2_/GA crosslinked polymer films is less intense and shifts toward higher temperatures. It can be inferred that the thermal stability is enhanced as a result of the combined influence of SiO_2_ and TiO_2_ fillers, along with the crosslinking interactions between PVA and GA. The TGA findings demonstrated that incorporating SiO_2_ and TiO_2_ into PVA/GA-based nanocomposites, PVA/5%SiO_2_/GA and PVA/5%SiO_2_/1%TiO_2_/GA, significantly improves their thermal resistance.

### 2.7. Contact Angle of the Membrane

**Figure 7:**
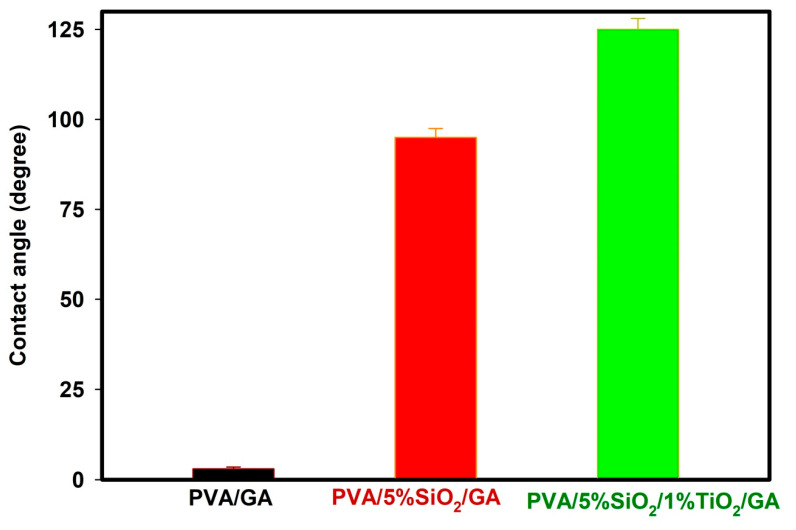
Contact angles of PVA/GA, PVA/5%SiO_2_/GA, and PVA/5%SiO_2_/1%TiO_2_/GA nanocomposites.

The water permeability of a membrane is significantly influenced by the wettability of its samples. Typically, increased hydrophilicity is associated with enhanced water permeability [36]. The contact angle of a membrane is a key parameter in characterizing its surface wettability, which directly affects its interactions with liquids. It is the angle formed between a liquid droplet and the membrane surface at the three-phase boundary where the liquid, solid, and air meet. This measurement offers valuable insight into determining whether a membrane is hydrophilic or hydrophobic. Hydrophilic membranes, characterized by a contact angle of less than 90°, exhibit an affinity for water, enabling improved liquid spreading and enhanced permeability. In contrast, hydrophobic membranes, with a contact angle greater than 90°, repel water and demonstrate limited wetting properties. These repel water, making them more resistant to wetting and are typically useful for gas separation or filtration applications. The wettability of the treated PVA/GA surface was inspected through contact angle measurements (Figure 7). It was observed that modifying PVA/GA by SiO_2_ and TiO_2_ (PVA/5%SiO_2_/GA and PVA/5%SiO_2_/1%TiO_2_/GA) had a significant effect on the contact angle generated by the treated PVA/GA. Pure PVA/GA exhibits a contact angle of 0°, as it is highly susceptible to instant wetting and swelling upon contact with water droplets. This behavior is attributed to the capillary effect and the presence of numerous hydrophilic hydroxyl groups in PVA, rendering PVA/GA superhydrophilic [61]. Alternatively, treating PVA/GA with 5%SiO_2_ (PVA/5%SiO_2_/GA) raised the contact angle to 95°, while treating PVA/GA with 5%SiO_2_ and 1%TiO_2_ increased the contact angle to 125°. SiO_2_ with TiO_2_ exhibit superhydrophobic properties, showing minimal hydrophilic characteristics. As a result, incorporating 5%SiO_2_ and 3%TiO_2_ into PVA/GA enhances the contact angle measurement. The rise in contact angles across all treated PVA/GA samples suggests a reduced water affinity, highlighting an improvement in their hydrophobic nature. The findings revealed that the loaded PVA/GA by 5%SiO_2_/1%TiO_2_ exhibits significant hydrophobic properties. In conclusion, the advanced treatment seems to make PVA/GA hydrophobic, allowing water droplets to remain stable on its surface (Figure 7). The treatment of PVA/GA with 5%SiO_2_/1%TiO_2_ created a layer with particles on the PVA/GA surface, improved the surface roughness, and improved the surface hardness. The contact angle of PVA/GA treated with 3%SiO_2_ and 5%SiO_2_/1%TiO_2_ was enhanced primarily because of reduced surface tension and roughness in the modified formulation. A key factor in the treated surface’s ability to resist aqueous liquids is its low surface tension, which often plays a critical role in such interactions [4]. This result may be due to the effect of the presence of SiO_2_ with TiO_2_, which improves the hardness and roughness of the formulation and increases the contact angle. On the other hand, because GA/PVA contains many hydroxyl groups in its structure, it exhibits a higher degree of hydrophilicity. Therefore, GA/PVA has a lower contact angle than other samples containing very few or no hydroxyl groups. When silica was added to PVA (PVA/5%SiO_2_), the contact angle increased significantly (95°). This behavior is mainly attributed to the reduced number of hydroxyl groups on the PVA surface, which can react with the silanol groups in the silica (condensation reaction), as shown in the results of Fourier transform infrared (FTIR) and swelling capacity (Figure 2) [62]. As shown in Figure 7, the contact angle of the films increased after the addition of 1% nano-titanium dioxide (PVA/5%SiO_2_/1%TiO_2_/GA), this occurs as a result of interactions between SiO_2_ and TiO_2_ or their interaction with the polymer matrix. The dispersed nanoparticles act as a physical barrier, limiting water penetration into the polymer network. This can reduce the overall water absorption of PVA. In addition to reducing polymer chain mobility, the presence of these nanoparticles can alter the structure of the polymer matrix, making it less susceptible to water absorption by reducing the availability of polar functional groups. This suggests that the nanoparticles (SiO_2_ and TiO_2_ nanoparticles) are evenly distributed within the polymer core and well-associated with the GA/PVA chains [63,64,65].

### 2.8. Soil Resistance

**Figure 8:**
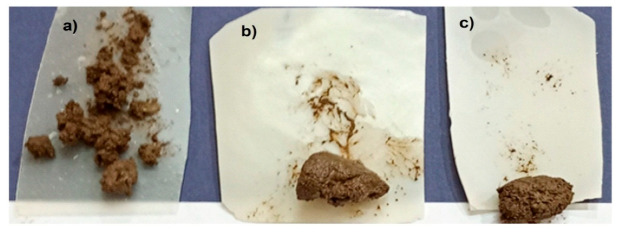
Camera photos of soil resistance of (**a**) PVA/GA, (**b**) PVA/5%SiO_2_/GA, and (**c**) PVA/5% SiO_2_/1%TiO_2_/GA nanocomposites.

To evaluate the self-cleaning capability of the treated PVA/GA, soil resistance was analyzed. Unlike the treated variants, untreated PVA/GA fails to repel the soil solution, leaving soil particles to adhere to its surface, as depicted in Figure 8. These particles create stubborn stains that are difficult to eliminate. In contrast, treated PVA/GA samples (PVA/5% SiO_2_/GA and PVA/5% SiO_2_/1% TiO_2_/GA) demonstrate remarkable soil resistance, with no residual soil observed after washing. However, untreated PVA/GA retains soil particles even post-cleaning, as illustrated in Figure 8. The PVA/5%SiO_2_/1%TiO_2_/GA nanocomposite is more resistant to the soil than PVA/5%SiO_2_/GA.

### 2.9. Application of PVA/5%SiO_2_/1%TiO_2_/GA in Sand Soil Stabilization

**Figure 9:**
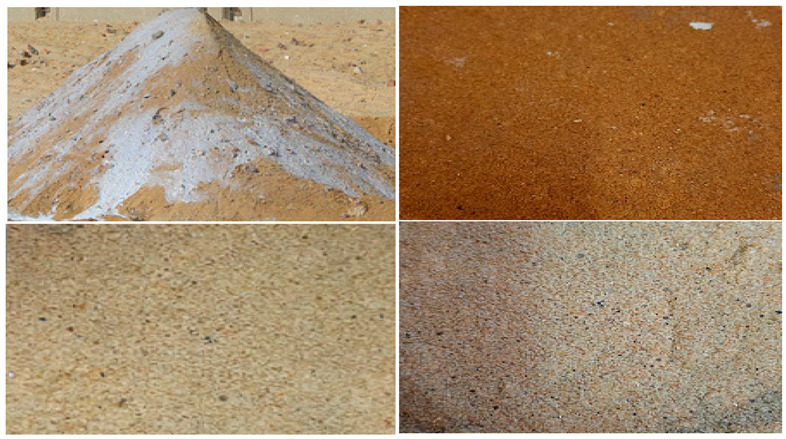
Photographic sample of sandy soil stability using PVA/5%SiO_2_/1%TiO_2_/GA with sand.

As shown in Figure 9 and Table 1, the application results of PVA/5%SiO_2_/1%TiO_2_/GA show that it is an effective soil stabilizer in enhancing the erosion resistance of surface soil and decreasing or preventing water absorption. Therefore, PVA/5%SiO_2_/1%TiO_2_/GA holds significant potential for widespread use in sand stabilization due to its effective stabilization mechanisms and cost-efficiency. PVA/5%SiO_2_/1%TiO_2_/GA solution is sprayed or mixed on the surface of sandy soil to stabilize it on sloping or semi-flat surfaces. PVA/5%SiO_2_/1%TiO_2_/GA solution can quickly diffuse into the pores between sand grains on the soil surface due to the relatively large inter-spaces between sandy soil particles. PVA/5%SiO_2_/1%TiO_2_/GA can enhance the soil mechanical properties by increasing the interaction between soil particles due to the adhesive property of PVA/5%SiO_2_/1%TiO_2_/GA, which leads to increased adhesion between sandy soil particles and closing the spaces between them [66]. As shown from the results in Table 1, the data of surface hardness, abrasion, water absorption, and contact angle are presented for the comparison between PVA/GA/sand and PVA/5%SiO_2_/1%TiO_2_/GA/sand. The results show that the PVA/5% SiO_2_/1%TiO_2_/GA/sand formulation improved and increased in all properties, with higher hardness, lower abrasion, and lower water absorption than the PVA/GA/sand formulation. The data indicated that the use of PVA/5% SiO_2_/1%TiO_2_/GA as a stabilizer for sandy soil significantly improved the hardness, abrasion, water absorption, and self-cleaning at 4phr addition levels, which are considered ideal addition levels.

## 3. Experimental and Techniques

### 3.1. Materials

Poly(vinyl alcohol) (PVA) [CH_2_CH(OH)], with an average molecular weight ranging between 17,000–18,000 Daltons and approximately 87–89% hydrolysis level, along with hydrochloric acid (HCl, 36.5% concentration) and glutaraldehyde, were procured. TiO_2_ nanopowder with a purity of 99.7% and particle sizes below 25 nm, as well as SiO_2_ of 10–20 nm diameter and 99.5% purity, were sourced from Sigma Aldrich Chemie GmbH (Steinheim, Germany). In addition, other chemicals such as NaOH, HCl, KCl, and citrate phosphate solutions were classified as buffers and were used as received.

### 3.2. Preparation of Hydrophobic PVA/SiO_2_/GA and PVA/SiO_2_/TiO_2_/GA Nanocomposites

To prepare a 5% by weight PVA solution, 50 g of PVA were mixed into 1000 mL of distilled water. The mixture was continuously stirred at 90 °C for 2 h until the PVA fully dissolved. The solution mixture underwent cooling to 25 °C; PVA/1%, 3%, and 5%SiO_2_/GA and PVA/5%SiO_2_/1% TiO_2_/GA nanocomposites were synthesized. In addition to preparing a PVA/GA solution, a 1 wt% solution of glutaraldehyde (GA) was added directly to the viscous polymer mixture for crosslinking reaction. Appropriate amounts of SiO_2_ nanopowder (1, 3, and 5 wt% of PVA solution), HCl and H_2_O (SiO_2_:HCl:H_2_O = 1:0.001:20 in molar ratio) were incorporated into the PVA solution. This mixture was then stirred continuously for 30 min. To the previously prepared PVA/5% SiO_2_ solution, 1% TiO_2_ nanopowders were incorporated and stirred continuously for 30 min. The reaction mixtures, including PVA/1%, 3%, and 5% SiO_2_/GA, PVA/5% SiO_2_/1% TiO_2_/GA, and PVA/GA, were thoroughly mixed and then subjected to ultrasonication at 70 W for 1 h to ensure complete dispersion of the SiO_2_ and TiO_2_ nanopowders. Ultrasonication enables the creation of diverse nanocomposites with customizable sizes and structures. Following this, the mixtures of PVA combined with 1%, 3%, and 5% SiO_2_/GA, PVA with 5% SiO_2_ and 1% TiO_2_/GA, as well as PVA/GA, were allowed to cool to ambient temperature. These mixtures were then transferred into plastic containers for further analysis and practical use.

### 3.3. Preparation of Buffer Solutions with Different pH Values

The swelling process of samples at different pH values for individual solutions was studied as follows: pH 2 solutions were obtained by mixing 0.1 M hydrochloric acid (4.2 mL of 36.5% hydrochloric acid diluted to a volume of 500 mL with distilled water) and 0.1 M potassium chloride (7.46 g + water per liter); pH 2 solutions were obtained by mixing 10.6 mL of hydrochloric acid with 89.4 mL of potassium chloride; pH 4 and pH 6 solutions were obtained by mixing 0.1 M citric acid (19.21 g/L, water weight: 192.1) and 0.1 M sodium citrate dihydrate (29.4 g/L, water weight: 294.0); solutions with pH 4 and 6 were obtained by mixing citric acid and sodium citrate solutions in the ratios 31:19 and 7:43 mL, respectively. The final volume of 100 mL was obtained by adding deionized water to achieve the desired pH values of 7, 8, 10, and 11, using the following solutions: 0.1 M disodium hydrogen phosphate (14.2 g/L), 0.1 M hydrochloric acid, and 0.1 M sodium hydroxide (0.4 g/L). The following ratios of the three above compounds were mixed to obtain the desired pH solutions: 75.6:24.4:0 mL for pH 7, 95.5:4.49:0 mL for pH 8, 95.5:4.50:0 mL for pH 10, and 96.5:34.7:0 mL for pH 11 [51].

### 3.4. Methods and Measurements

Gel content measurement: To isolate the insoluble components of the hydrogels, specifically the crystalline portion, the samples were immersed in water at 60 °C for 24 h. Afterwards, they were removed, rinsed with hot water to eliminate the soluble fraction, dried, and weighed. The gel content was calculated using the following formula [67]:

$$Gel\;content\;\left(\%\right)=\left(\frac{W_{1}}{W_{2}}\right)\times 10$$

where *W*_1_= weight of the dried gel post-soaking (g) and *W*_0_ = gel’s original weight (g).

Water absorption measurement: The dried sample was first weighed and then submerged in water for 24 h to achieve absorption equilibrium. At specific intervals, the swollen hydrogel was removed from the water, and any surface moisture was carefully blotted away using filter paper. The swelling degree, in grams of water per gram of polymer (g/g), was determined using the formula below [67]:

$$Swelling\;capacity\left(\frac{g\;wet}{g\;dry}\right)=\left(\frac{W_{t}-W_{0}}{W_{0}}\right)$$

where *W_t_* = swollen gel weight at time t (g) and *W*_0_ is the original weight of the gel in its dry form (g).

X-ray diffraction. XRD patterns was recorded using a Shimadzu diffractometer (Tokyo, Japan), XRD-6000 x-ray diffraction spectrometer with a copper target (λ = 1.542 A^o^), at an operating voltage of 40 kV and an electric current of 30 mA. The XRD patterns were systematically obtained within the 10–90° 2θ angular range, with a scanning rate of four steps per minute.

High-resolution transmission electron microscope (TEM): The particle size and shape of the generated SiO_2_ and TiO_2_ nanoparticles were measured using a high-resolution transmission electron microscope (HRTEM, JEM2100, Jeol, Tokyo, Japan).

Scanning electron microscopy (SEM): A JEOL JSM scanning electron microscope, functioning at 30 kV, was employed to examine the surface of the samples. Prior to this analysis, the samples underwent coating with a thin layer of gold to minimize charging effects while the samples underwent the microscopy process.

Infrared spectroscopic analysis (FTIR): Infrared spectroscopy was performed at an ambient temperature range of 400–4000 cm^−1^ utilizing a Bruker Vertex 70 v (Billerica, MA, USA) vacuum Fourier transform infrared (FTIR) spectrometer.

Surface hardness: The hardness was measured according to the ASTM D2240 [68] standard using a model 306-type A durometer for both hard and soft plastics.

Thermogravimetric analysis (TGA): Achieved utilizing a Shimadzu TGA-50 framework (Kyoto, Japan), with the temperature gradually increased from 25 to 600 °C at a rate of 10 °C/min. The analysis was performed under a steady nitrogen flow of 20 mL/min.

Contact angle: The contact angle between water and samples was quantified utilizing a Biolin Scientific Theta (Biolin Scientific, Gothenburg, Sweden) lite optical tensiometer, employing the static drop method.

Soil resistance: Following the AATCC-130–2015 standard [69], soil spot resistivity was evaluated by substituting corn oil with water. A solution was prepared by combining 5 g of soil with 5 mL of water. Three test specimens, each measuring between 50 cm and 100 cm, were used to assess soil spots. Each sample was secured on a glass slide at a 45-degree angle with clamps. Soil impedance was measured at five identical spots for 20 s, comparing the results to standardized spot references.

Abrasion test: An AP. 40 (Maschinebau Gmbh Raueustein Thuringeu, Raueustein, Germany) was used, which has the following specification: cylinder diameter: 150 mm, cylinder length: 560 mm, cylinder speed: 40 rpm, specimen diameter: 16 mm, specimen clamping height: 15 mm, abrasion path: 40 m.

Loss in the mass was calculated according to the following equation [70]:

$$Mass\;loss\;\left(mg\right)per\;revolution=\left(\frac{W_{i}}{W_{f}}\right)\times 10$$

where W_i_ is the original mass of sample (g), W_f_ is the final mass of sample (g), and n is the number of revolution (84).

Hardness test: Surface hardness was measured using ASTM D 2240 specifications, model 306 L type A, D durometer for soft and hard plastic.

## 4. Conclusions

The nanocomposites of PVA/5%SiO_2_/GA and PVA/5%SiO_2_/1%TiO_2_/GA were prepared by a simple two-step protocol via sol–gel process and adding GA as a crosslinking agent. It is worth mentioning that compared with PVA/GA hydrogel, the physical properties of PVA/5%SiO_2_/GA and PVA/5%SiO_2_/1% TiO_2_/GA nanocomposites are increased several times and have very low swelling capacity. The experimental study led to the following conclusions:

1. The optimum miscibility and high gel content (%) formation was found to be at PVA/5%SiO_2_/1%TiO_2_/GA.
2. The PVA/GA water absorption declined from 3.1 g/g to 0.07 g/g due to the presence of SiO_2_ and TiO_2_ at 5%SiO_2_ and 1%TiO_2_ (PVA/5% SiO_2_/1% TiO_2_/GA), and this ratio is the best ratio as PVA/GA turns from hydrophilic to hydrophobic.
3. Compared to the PVA/GA IR spectra, the peak of -OH group stretching vibrations shifted to a lower wavenumber. This shift suggests that a significant portion of the hydroxyl groups in PVA interacted with the decomposition products of SiO_2_ and TiO_2_, leading to the formation of hydrogen bonds.
4. The TGA findings demonstrated that incorporating SiO_2_ and TiO_2_ into PVA/GA-based nanocomposites, PVA/5%SiO_2_/GA and PVA/5%SiO_2_/1%TiO_2_/GA, significantly improves their thermal resistance.
5. The treatment of PVA/GA with 5%SiO_2_/1%TiO_2_ created a layer with particles on the PVA/GA surface, improved the surface roughness, and increased the surface hardness and contact angle.
6. The integrated properties of the prepared PVA/5% SiO_2_/1% TiO_2_/GA nanocomposites make them potential alternatives for sandy soil stabilization and construction works.

## Figures and Tables

**Table 1:** Mechanical properties tests of PVA/GA/sand and PVA/5%SiO_2_/1%TiO_2_/GA/sand.

Samples	Surface Hardness (Shore D)	Abrasion Test	Water Absorption (g/g)	Contact Angle (θ)	Loading on Compaction (at 200 Kg/cim ^2^)
PVA/GA/sand	71	8	3.4	0	7%
PVA/5% SiO_2_/1% TiO_2_/GA/sand	87	2	0.01	125	3%

## Data Availability

Data are contained within the article.
